# Renal function protection and the mechanism of ginsenosides: Current progress and future perspectives

**DOI:** 10.3389/fphar.2023.1070738

**Published:** 2023-02-06

**Authors:** Meiling Fan, Xintian Lan, Qunling Wang, Mengyao Shan, Xiaoxue Fang, Yegang Zhang, Donglu Wu, Haoming Luo, Wenyi Gao, Difu Zhu

**Affiliations:** ^1^ The Affiliated Hospital of Changchun University of Chinese Medicine, Changchun, China; ^2^ School of Pharmacy, Changchun University of Chinese Medicine, Changchun, China; ^3^ Key Laboratory of Effective Components of Traditional Chinese Medicine, Changchun, China; ^4^ School of Clinical Medical, Changchun University of Chinese Medicine, Changchun, China

**Keywords:** ginsenoside, kidney, nephropathy, animal model, mechanism

## Abstract

Nephropathy is a general term for kidney diseases, which refers to changes in the structure and function of the kidney caused by various factors, resulting in pathological damage to the kidney, abnormal blood or urine components, and other diseases. The main manifestations of kidney disease include hematuria, albuminuria, edema, hypertension, anemia, lower back pain, oliguria, and other symptoms. Early detection, diagnosis, and active treatment are required to prevent chronic renal failure. The concept of nephropathy encompasses a wide range of conditions, including acute renal injury, chronic kidney disease, nephritis, renal fibrosis, and diabetic nephropathy. Some of these kidney-related diseases are interrelated and may lead to serious complications without effective control. In serious cases, it can also develop into chronic renal dysfunction and eventually end-stage renal disease. As a result, it seriously affects the quality of life of patients and places a great economic burden on society and families. Ginsenoside is one of the main active components of ginseng, with anti-inflammatory, anti-tumor, antioxidant, and other pharmacological activities. A variety of monomers in ginsenosides can play protective roles in multiple organs. According to the difference of core structure, ginsenosides can be divided into protopanaxadiol-type (including Rb1, Rb3, Rg3, Rh2, Rd and CK, etc.), and protopanaxatriol (protopanaxatriol)- type (including Rg1, Rg2 and Rh1, etc.), and other types (including Rg5, Rh4, Rh3, Rk1, and Rk3, etc.). All of these ginsenosides showed significant renal function protection, which can reduce renal damage in renal injury, nephritis, renal fibrosis, and diabetic nephropathy models. This review summarizes reports on renal function protection and the mechanisms of action of these ginsenosides in various renal injury models.

## 1 Introduction

Nephropathy is a general term for kidney-related diseases, which refers to changes in the structure and function of the kidney caused by various factors, resulting in pathological damage to the kidney, abnormal blood or urine components, and other diseases. The main clinical manifestations of nephrosis are hematuria, albuminuria, edema, hypertension, anemia, lower back pain, oliguria, and other symptoms. Nephropathy can be divided into glomerular, renal tubular, renal interstitial, and vascular diseases according to the main components of the disease. Chronic renal diseases may eventually lead to chronic renal failure. Renal disease can be divided into acute renal injury and chronic renal failure according to the degree, reversibility, and time of occurrence of renal function damage. Nephropathy can be divided into primary and secondary nephropathy. Primary nephrosis includes immune reaction-mediated nephritis, infectious diseases of the urinary system, renal vascular diseases, renal stones, renal tumors, and congenital nephrosis. Secondary nephropathy can be induced by tumors, metabolism, autoimmune diseases, and other diseases, which are also observed with various drugs, toxins, and other damage to the kidney. Acute kidney injury (AKI) is a prevalent critical renal disease associated with a high risk of death in hospitalized patients. Data from 2015 showed that both the incidence of AKI and mortality after AKI in inpatients exceeded 20% and the prognosis of AKI did not improve significantly (Mehta, et al. 2015). Recent studies have confirmed that renal function cannot completely recover or even require long-term renal replacement therapy in some patients with AKI. AKI eventually progresses to chronic kidney disease (CKD) or end-stage renal disease (ESRD) (Bucaloiu, et al. 2012). Nephritis is one of the most common kidney-related diseases. In clinical practice, glomerulonephritis (GN) is usually referred to as nephritis, which is an immune reaction disease (Coresh, et al. 2003). Renal fibrosis (RF) is a pathophysiological change that is a progressive process of renal function, deteriorating from health to injury until loss of function occurs. RF is also involved in the terminal pathway in CKD and nephritis. The therapeutic effects of CKD and nephritis are closely related to the degree of RF (Gewin, et al. 2017). Diabetic nephropathy (DN) is another major category of kidney-related disease. DN manifests as glomerulosclerosis, caused by diabetic microangiopathy. Once DN develops, most patients develop ESRD (Reutens and Atkins, 2011). Nephropathy, especially CKD and DN, has become a global public health issue with increasing annual prevalence. These urgent problems have prompted researchers to develop more drugs and methods for the treatment of nephropathy.

In recent years, a class of chemical drugs, including selective endothelin receptor antagonists and phosphodiesterase inhibitors, have been used to improve renal function because of their effects on reducing fasting blood glucose and glycosylated hemoglobin levels, as well as anti-inflammatory and anti-fibrosis effects. However, through the summary of previous clinical experience, it was found that some drugs are very easy to produce a variety of adverse reactions in the process of use, and can induce urinary tract infections, thus reducing the efficiency of clinical treatment. After a series of research experiments medical experts found that Chinese medicine plays an important role in the treatment of the disease, and Chinese medicine can regulate blood sugar as well as lipid metabolism to minimize kidney damage, thus creating favorable conditions for promoting early recovery.

**FIGURE 1:**
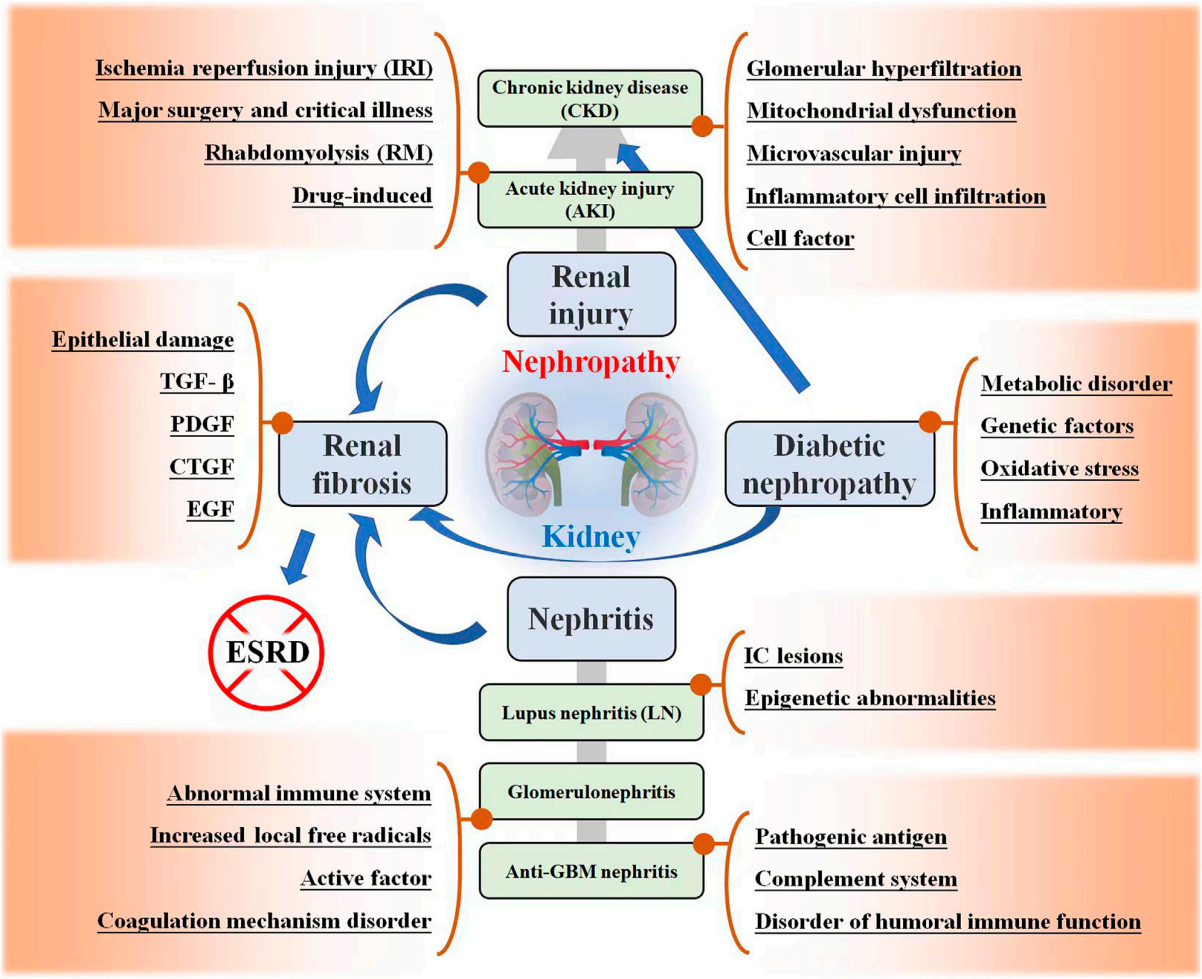
Kidney related diseases, including renal injury, renal fibrosis (RF), nephritis, and diabetic nephropathy (DN). The possible mechanisms and development trends of these diseases are listed in the figure.

Ginsenosides are the main active components of the traditional Chinese herbal medicine Panax ginseng C. A. Mey (Song, et al. 2022). At present, nearly 200 ginsenosides have been isolated and identified from the roots, stems, leaves, flower buds, and berries of Panax ginseng C. A. Mey. (Zhao, et al. 2022a). Ginsenosides can be divided into three classes according to their aglycone structure: protopanaxadiol (PPD), protopanaxatriol (PPT), and oleanolic acid. The PPD types mainly include ginsenosides Rb1, Rd, Rg3, Rh2, CK, and F2, etc., PPT types mainly include ginsenosides Re, Rf, Rg1, Rg2, Rh1, and F1, etc., and oleanolic acid types mainly include ginsenoside Ro (Hou, et al. 2021, Liu, et al. 2022). Modern pharmacological studies have shown that ginsenosides have neuroprotective (Zarneshan, et al. 2022), anti-aging (Meng, et al. 2022), anti-oxidant (He, et al. 2022a), anti-inflammatory (Xu, et al. 2022), and anti-cancer (Zhao, et al. 2022b), effects (Figure 1). Nowadays, many renal protective drugs have been widely used, but some of them have potential adverse effects. Monomers extracted from traditional Chinese herbal medicines have attracted considerable attention as effective and safe substitutes for kidney diseases (Gao, et al. 2017). Ginsenoside has been proven to have significant renoprotective effects and can be used as an antioxidant, anti-inflammatory, and anti-apoptotic agent. This review summarizes the protective effects and molecular mechanisms of ginsenosides in various types of kidney diseases.

## 2 Nephropathy

Nephropathy is a general term for common kidney diseases that seriously endanger human health, including different types of renal injury, renal failure, nephritis, renal fibrosis, and DN. The pathogenesis of kidney disease is complex and often involves multiple mechanisms, as shown in Figure 1. Different types of kidney diseases may also interact and progress. Here, the characteristics of these kidney-related diseases are introduced.

### 2.1 Renal injury

Acute kidney injury (AKI) is a common clinical emergency characterized by a rapid decline in renal function, which eventually leads to acute renal failure (ARF) and other organ failures. The etiology of AKI is complex and diverse and can be divided into prerenal, renal, and postrenal according to the anatomical location of the etiology. Prerenal AKI refers to a progressive decrease in blood flow perfusion in the renal parenchyma due to various causes, leading to a progressive decrease in the glomerular filtration rate (GFR). Renal AKI refers to renal parenchymal damage caused by a variety of factors, including the unrelieved renal ischemia of pre-renal AKI and damage to glomeruli, renal tubules, renal interstitium, and renal microvessels. Postrenal AKI refers to urinary tract obstruction caused by many factors and can be generally divided into intrarenal, extrarenal, and urethral obstruction (Chen and Guo, 2019). In recent years, the incidence of AKI has been increasing, and the incidence in hospitalized patients which has reached 1%–5%, has been growing rapidly. Once AKI occurs, the fatality rate of patients increases significantly, and the death rate of severe cases is >50% (Weiyun, et al. 2019).

Chronic kidney disease (CKD) is a disease of chronic renal insufficiency caused by various primary or secondary causes of renal injury and is characterized by chronic glomerular and renal tubule injury. CKD is defined as persistent urinary abnormalities, structural abnormalities, or impaired renal function, suggesting loss of functional nephrons (Romagnani et al. 2017; Diwan et al. 2018). The basic clinical manifestations of CKD include proteinuria, hematuria, hypertension, and edema. CKD pathogenesis mainly involves immune responses, cytokines, inflammatory mediators, and hemodynamic abnormalities.

Previously, it was believed that the kidney had a strong compensatory capacity, and the renal function of patients with AKI could recover better. However, recent studies have confirmed that the renal function of a considerable number of AKI patients that cannot be completely recovered, may even require long-term renal replacement therapy, eventually progressing to CKD or ESRD (Lo et al. 2009; Chawla et al. 2014). The important pathogenic mechanisms of AKI progression to CKD include glomerular hyperfiltration and hypertrophy, mitochondrial dysfunction (Zhan et al. 2013), cell infiltration and secretion of bioactive molecules (Venkatachalam et al. 2010), reduction in renal capillary density, and tubulointerstitial fibrosis (Chawla and Kimmel, 2012). Cytokines such as ET-1 (López-Farré, Gómez-Garre, et al. 1991), TGF- β (Gentle, et al. 2013), serum galectin-3 (Calvier, et al. 2013), and HIF (Nangaku, et al. 2013), play a role in these pathways.

Some chemotherapeutic drugs and chemical reagents for diagnosis and treatment may lead to drug-induced AKI. As a highly effective and broad-spectrum anticancer drug, the renal transport of cisplatin (CDDP) is regulated by proximal tubular transporters, which accumulate in proximal tubular epithelial cells, causing inflammation, injury, and cell death. More than a third of the patients receiving CDDP treatment suffer from nephrotoxicity, manifested as AKI, loss of serum sodium and magnesium, and dysfunction of the urine concentration (Miller, et al. 2010). AKI is the main complication of CDDP-induced nephrotoxicity.

### 2.2 Nephritis

In a narrow sense, nephritis refers to glomerulonephritis, which is generally referred to as nephritis in clinical practice. Broadly, nephritis includes pyelonephritis, glomerulonephritis (GN), and tubulointerstitial nephritis. Pyelonephritis is an inflammation of the renal pelvis and renal parenchyma caused by pathogenic microorganisms and is often accompanied by lower urinary tract infections. Glomerulonephritis, a disease caused by an immune reaction, is commonly referred to as nephritis and is mainly located in the glomerulus. Interstitial nephritis, also known as tubulointerstitial nephritis, is a clinical-pathological syndrome of acute and chronic renal tubulointerstitial damage caused by various factors.

Anti-glomerular basement membrane (GBM) nephritis is an autoimmune disease associated with the GBM antibody (Lahmer and Heemann, 2012). Nephritis belongs to type one rapidly progressive glomerulonephritis, the pathological classification of which is crescentic nephritis. Most patients have an acute onset, rapid progression, and poor prognosis (Fernandes, et al. 2016). Most untreated patients die of acute renal failure or pulmonary hemorrhage. The onset of the disease is characterized by rapid progressive nephritis syndrome.

Lupus nephritis (LN) is an immune injury caused by systemic lupus erythematosus (SLE) in different pathological kidney types. The clinical manifestations of LN are mostly similar to those of nephrotic syndrome or chronic glomerulonephritis, with edema, hematuria, proteinuria, hypertension, fever, rash, and other symptoms (Yung et al. 2017; Lin et al. 2019). LN is one of the main complications and lethal factors of SLE. LN pathogenesis mainly includes immune complex lesions (Anders and Fogo, 2014) and epigenetic abnormalities (Dieker, et al. 2007). In addition, abnormalities in the complement system (Hristova and Stoyanova, 2017), sexual hormone disorders (Feng, et al. 2010), and environmental effects are also related to the occurrence and development of LN.

### 2.3 Renal fibrosis

Renal fibrosis (RF) is a pathological result of long-term or repeated renal injury caused by single or multiple factors, the main pathological feature of which is excessive deposition of the extracellular matrix. The microscopic manifestation of RF is fibrosis of intrinsic renal cells, which is essentially the necrosis of intrinsic renal cells due to damage (Ke, et al. 2015). RF is the terminal stage of several chronic kidney diseases. It is a pathological process characterized by leukocyte infiltration, apoptosis, necrosis of renal tubular cells, the proliferation of tubulointerstitial fibroblasts, and deposition of extracellular matrix (Liu, 2010). Studies have shown that in addition to epithelial damage, the mechanism of RF is related to growth factors, such as transforming growth factor β (TGF-β) (Liu 2006), platelet-derived growth factor (Ostendorf, et al. 2014), connective tissue growth factor (Burns, et al. 2006), and epidermal growth factor (Stangou, et al. 2009). Renal fibrosis can be divided into two stages depending on the extent of damage to the intrinsic cells of the kidney and whether they can be repaired namely the reversible stage of fibrosis formation and progression, and the scar formation stage. The treatment of the first stage is of great significance for the rehabilitation of kidney disease and reversal of renal failure, which should be urgently addressed by doctors and patients. In the second stage, although it is possible to prevent the progression of renal fibrosis, it is difficult to repair scarred renal tissue (Humphreys, 2018).

### 2.4 Diabetic nephropathy

More than 30% of patients with diabetes suffer from DN, which is the main cause of morbidity and mortality. DN is characterized by early microalbuminuria, which gradually develops into massive albuminuria and progressive renal insufficiency, and finally forms ESRD. Unfortunately, once ESRD develops, the 5-year survival rate of patients is usually less than 20% (Natesan and Kim, 2021). The main pathological features of DN include glomerulosclerosis, tubulointerstitial fibrosis, and renal vascular disease. The pathogenic factors and pathogenesis are complex and include metabolic disorder (Xu, et al. 2018; Chen, et al. 2020), genetic factors (Wu, et al. 2021a), oxidative stress (Stehouwer, 2004; Sagoo and Gnudi, 2018), and inflammatory mechanisms (Wada and Makino, 2013), etc*.*

## 3 Application of ginsenosides in kidney related diseases

**FIGURE 2:**
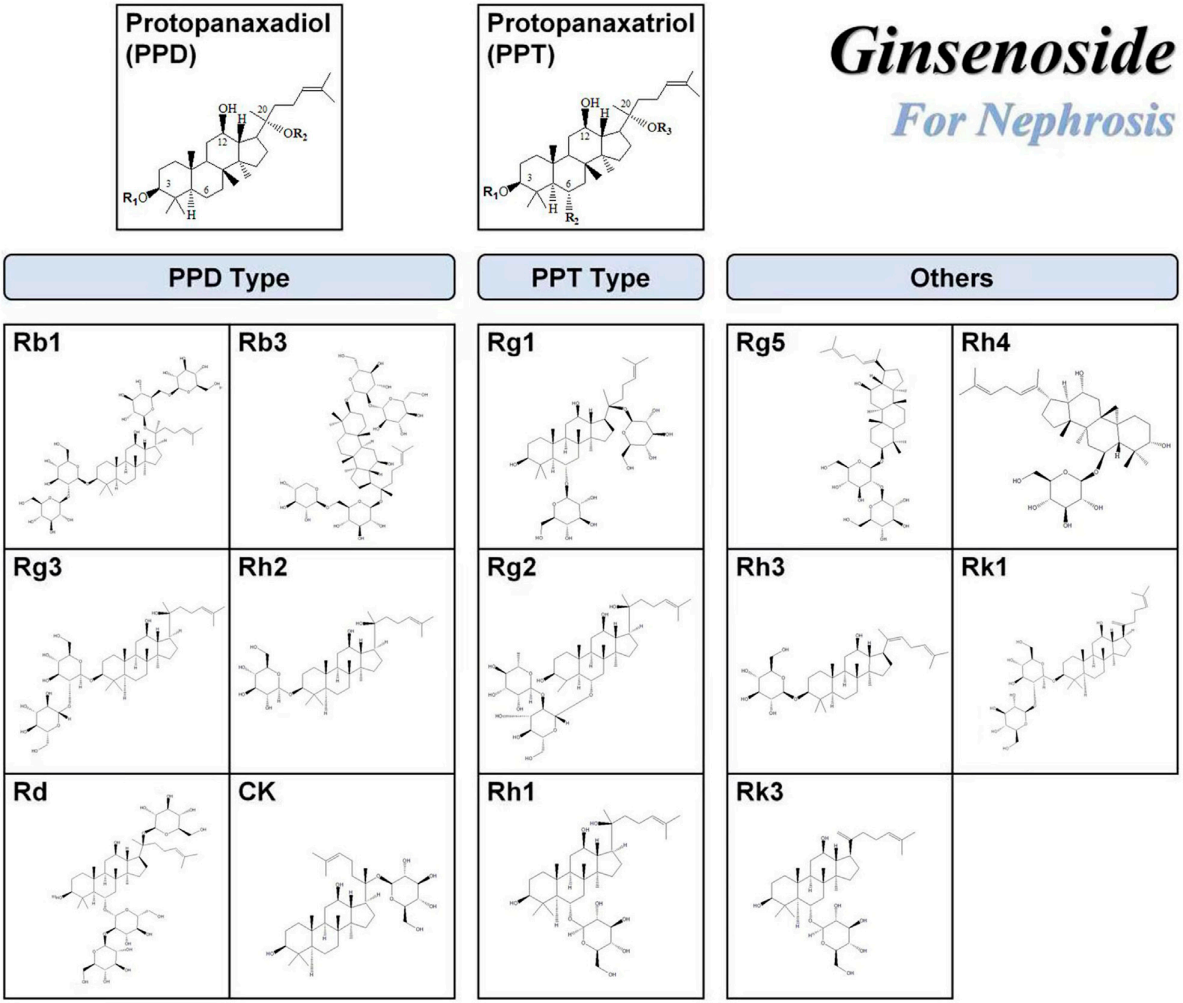
Chemical structures of ginsenosides with renal function protection. The chemical structures of the ginsenosides included in this publication were drawn using the ChemDraw program.

Ginsenosides are usually composed of 30 carbon atoms, with a 4-ring steroid structure and a sugar group. The history of separating ginsenosides from plants (e.g., ginseng, Panax notoginseng, American ginseng) can be traced back to 1854. More than 100 types of ginsenosides have been identified and successfully classified (Shin, et al. 2015). Each ginsenoside has at least two (C-3 and C-20) or three (C-3, C-6, and C-20) hydroxyl groups, which are free or linked to monomers, disaccharides, or trimers in most cases (Shin and Oh, 2016). The variety of ginsenosides is due to the different positions and the number of glycosyl groups connected by triterpene saponins, stereoisomerism of ginsenosides, and variable side chains at the C-20 position. Furthermore, it has created a variety of active ingredients, including anti-cancer, anti-diabetes, anti-fatigue, anti-aging, liver protection, and for kidney protection (Yenisetti et al. 2016). According to the position of the sugar group at C-3 and C-6, ginsenosides are divided into three categories: protopanaxadiol (PPD)-type saponins, protopanaxatriol (PPT)-type saponins, and oleanolic acid-type saponins (others) (Qu, et al. 2009), as shown in Figure 2. Ginsenosides with a high content of ginseng, such as Re, Rg1, Rd, and Rb1, contain many sugar residues, which makes it impossible or difficult to be directly absorbed and utilized by the human body after ingestion. These ginsenosides usually need to be metabolized and converted into smaller molecules before being absorbed by the human body, which greatly affects their biological activities. After chemical or biological transformation, the main ginsenosides can be metabolized into rare ginsenosides such as Rg3, Rk1, Rg5, CK, Rk3, and Rh4. These rare saponins show higher pharmacological activity owing to the reduction of sugar residues linked to their molecular structure, increased hydrophobicity, and enhanced cellular penetration. In addition, a variety of ginsenosides have been shown to exert anti-cancer, anti-diabetic, anti-fatigue, anti-aging, hepatoprotective and renoprotective effects (Yenisetti et al. 2016). Numerous studies have shown that ginsenosides can protect the kidneys from damage through different pathways. For example, Ginsenoside Rb1 can treat acute kidney injury by activating the Nrf2/ARE pathway (Sun et al. 2012). The mechanisms of ginsenosides to alleviate renal diseases by improving glucolipid metabolism, inhibiting oxidative stress, anti-inflammation, anti-apoptosis, regulating autophagy, and anti-fibrosis are highlighted here, as shown in Table 1.

**TABLE 1:** The mechanism of action of various ginsenosides on different kidney related diseases.

Type	Ginsenosides	Nephropathy	Mechanism of drug action	References
PPD	Rb1	AKI	Activating the Nrf2/ARE pathway	Sun et al. (2012)
		CKD	Reducing oxidative stress and inflammation	Zhang et al. (2022)
		CKD	Inhibiting the Wnt/β-catenin pathway	Xu et al. (2017)
		CKD	Regulation of Akt-independent and AMPK-dependent mTOR signaling to inhibit autophagy	Zhou et al. (2019)
		RF	Downregulation of TGF-β1 expression	Xie et al. (2009)
		RF	Inhibiting Bip/eIF2α/CHOP signaling-mediated EMT	Ni et al. (2022)
		Type 2 DN	Regulating the expression of miR-3550 and further combining with Wnt/β-catenin signaling	Shao et al. (2019)
		Type 2 DN	Inhibiting aldose reductase activity	He et al. (2022a)
	Rb3	cisplatin-induced AKI	Regulating AMPK-/mTOR-mediated autophagy and inhibiting apoptosis	Xing et al. (2019)
		cisplatin-induced AKI	TGF-β-mediated mitochondrial apoptosis	Wu et al. (2021a)
	Rg3	D-galactose induced AKI	Inhibiting the renal oxidative stress caused by D-galactose, and at the same time activated the PI3K/AKT signaling pathway to attenuate the apoptosis of liver and kidney cells	Sun et al. (2013)
		LPS-induced AKI	Reducing the expression of NF-kB and iNOS proteins, and reduced the expression of COX-2 and HO-1 proteins	Kang et al. (2007)
		cisplatin-induced AKI	Regulation of PI3K/AKT and NF-κB-mediated apoptosis and inflammatory pathways	Zhang et al. (2021)
		cisplatin-induced AKI	Inhibiting NLRP3 by inhibiting apoptosis and autophagy	Zhai et al. (2021)
		cisplatin-induced AKI	Blocking the JNK-p53-caspase-3 signaling cascade	Han et al. (2016)
		cisplatin-induced AKI	Regulating inflammation and apoptosis	Park et al. (2015)
		Type 2 DN	Regulation of MAPK/NF-κB signaling pathway	Li et al. (2021)
		Type 2 DN	Inhibit inflammation	Zhou et al. (2020)
		Type 2 DN	Inhibiting oxidative stress and advanced glycation end product formation	Kang et al. (2010b)
		kidney cancer	Blockade of TRPM7 channel activity	Kim et al. (2011)
	Rd	cisplatin-induced AKI	Inhibiting free radical-mediated lipid peroxidation while inhibiting apoptosis	Yokozawa and Dong (2001)
		cisplatin-induced AKI	Inhibition of lipid peroxidation by free radicals	Yokozawa and Liu (2000)
		kidney cancer	Inhibiting TRPM7 channel activity	Kim et al. (2013)
	Rh2	cisplatin-induced AKI	Acting on a caspase-mediated pathway	Qi et al. (2019)
		Type 2 DN	Down-regulating discoid domain receptor 1	Shen et al. (2021)
	CK	Primary GN	Enhancing autophagy induction by inhibiting NLRP3 inflammasome activation in kidney tissue, macrophages, and bone marrow-derived dendritic cells, increasing SIRT1 expression, and triggering autophagy-mediated NLRP3 inflammasome inhibition	Wu et al. (2020)
		TIN	Inhibiting NLRP3 inflammasome initiation and mitochondria-related activation signaling in tubulointerstitial lesions	Hsu et al. (2020)
		Type 2 DN	Inhibiting NLRP3 inflammasome activation and NF-κB/p38 signaling pathway in diabetic nephropathy in high-fat diet/streptozotocin-induced diabetic mice	Song et al. (2018)
		Type 2 DN	Enhancing antioxidant capacity, reduced the damage of TGF-β1 to renal tissue	Shao et al. (2015)
	CK	kidney cancer	Regulating ROS and LNRNA THOR	Chen et al. (2021)
PPT	Rg1	AKI	Inhibition of sideroporosis in renal TEC by FSP1	Guo et al. (2022)
		D-galactose induced AKI	Preventing DNA damage by attenuating oxidative stress	Fan et al. (2016)
		CKD	Inhibiting NOX4-NLRP3 signaling in mice	Liu (2010)
		Anti-GBM RPGN	Increasing renal blood flow	Hattori et al. (1991)
		Anti-GBM RPGN	Activating NRF2 signaling	Guo et al. (2019)
		RF	Downregulation of TGF-β1 expression	Xie et al. (2008a)
		RF	Blocking TEMT by inhibiting the expression of TSP-1, thereby inhibiting the activation of TGF-β1	Xie et al. (2008b)
		RF	Inhibiting endoplasmic reticulum stress-induced apoptosis in rats after unilateral ureteral obstruction	Li et al. (2015)
		RF	Inhibiting TGF-β1-induced transdifferentiation of rat renal tubular epithelial cells	Xie et al. (2008c)
		RF	Inhibiting NOX4 and NLRP3 inflammasome activation in SAMP8 mice	Shen et al. (2020)
		RF	Regulating Klotho/TGF-β1/Smad signaling pathway	Li et al. (2018)
		Type 2 DN	Regulating the PI3K/AKT/FOXO3 pathway	Liu et al. (2021)
		Type 2 DN	Reducing the expression of TGF-β1 and the already mentioned inflammatory response factors in renal tissue	Ma et al. (2010)
		Type 2 DN	Reducing the expressions of TNF-α and MCP-1	Zhang et al. (2009)
		Type 2 DN	Reducing oxidative stress and inhibits TGF-β1/Smads signaling cascade in renal fibrosis in diabetic nephropathy rats	Du et al. (2018)
	Rg2 and Rh1	AKI	Blockade of LPS-TLR4 signaling reduced p38-STAT1 activation and NF-κB translocation, which in turn suppressed the transcription of inflammatory cytokines and mediators such as IFN-β, TNF-α, IL-1β, and iNOS	Huynh et al. (2020)
	Rh1	Type 2 DN	Regulation of AMPK/PI3K/Akt-mediated inflammatory and apoptosis signaling pathways	Su et al. (2021)
**Others**	Rg5	cisplatin-induced AKI	Inhibiting inflammation, oxidative stress and apoptosis	Li et al. (2016)
		cisplatin-induced AKI	Regulating inflammation and apoptosis	Park et al. (2015)
		Type 2 DN	Inhibiting NLRP3 inflammasome activation and MAPK signaling pathway in high-fat diet/streptzotocin-induced diabetic mice	Zhu et al. (2020)
	Rh3	kidney cancer	Inhibition of the JNK and ERK mitogen-activated protein kinase signaling cascades	Lee and Kang (2017)
	Rh4 and Rk3	cisplatin-induced AKI	anti-oxidation	Baek et al. (2006)

### 3.1 PPD type ginsenosides

The PPD type ginsenoside uses PPD as an aglycone. Generally, ginsenosides are divided into two configurations: 20 S)—PPD and 20 R)—PPD, according to the substitution of C-3 or C-20 hydroxyl groups of aglycones by different sugar groups. The structural formulas of common PPD-type ginsenosides are shown in Figure 1. The PPD ginsenoside group has been shown to have significant pharmacological activities, including ginsenosides Rb1, Rb2, Rb3, RC, Rg3, Rh2, Rd, and CK (Table 2). PPD has good biological activities such as antioxidant and anti-inflammatory (Yang et al. 2019). Several studies have reported the nephroprotective effects of PPD and related mechanisms.

**TABLE 2:** Pharmacological activity of PPD type ginsenosides.

Ginsenoside	Activity	References
Rb1	Neuroprotective, antioxidant, estrogen-like effects	Lee et al. (2003a)
		Park et al. (2005)
Rb2	Inhibition of tumor metastasis	Fujimoto et al. (2001)
Rb3	Antioxidative	Liu et al. (2002)
		Liu et al. (2003)
RC	Enhance immunity, anti-inflammatory effect	Berek et al. (2001)
		Surh et al. (2002)
Rg3	Anti-tumor, nerve protection, blood vessel protection, anti-platelet aggregation	Popovich and Kitts (2002)
		Keum et al. (2003)
Rd	Enhance immunity, antioxidant, protect cardiovascular and cerebrovascular	Berek et al. (2001)
		Liu et al. (2002)
		Liu et al. (2003)
Rh2	Antitumor	Bae et al. (2004)
		Kim and Jin (2004)
CK	Anti - heritable virus effect, anti—tumor	Lee et al. (1998)
		Lee et al. (2005)

#### 3.1.1 Ginsenoside Rb1

Ginsenoside Rb1 is one of the main active monomers of ginseng and has the ability to scavenge oxygen free radicals and thus has antioxidant activity. Ginsenoside Rb1 has been shown to inhibit renal ischemia-reperfusion injury and interstitial fibrosis and reduce renal cell apoptosis and oxidative damage. For instance, ginsenoside Rb1 upregulates Nrf2 and heme oxygenase-1 (HO-1) by activating the nuclear factor-related factor 2 (Nrf2)/ARE pathway, which in turn attenuates acute kidney injury caused by intestinal ischemia-reperfusion (Sun et al. 2012). In addition, the possible protective effects and mechanisms of ginsenoside Rb1 on oxidative damage and renal interstitial fibrosis in rats with unilateral ureteral obstruction (UUO) have been widely studied. Fan et al. found that ginsenoside Rb1 significantly inhibited renal interstitial fibrosis in UUO rats by down-regulating TGF-β1 (Xie et al. 2009) expression or ihibiting Bip/eIF2α/CHOP signaling-mediated EMT (Ni et al. 2022). There are reports that ginsenoside Rb1 treats acute kidney injury by activating the Nrf2/ARE pathway which acts against oxidative stress (Sun et al. 2012). Ginsenoside Rb1 can further prevent autophagy by inhibiting the Wnt/β-catenin pathway (Xu, et al. 2017) or by regulating Akt-independent (cell proliferation and survival) and AMPK-dependent mTOR signaling-involved in cell survival under energy stress (Zhou et al. 2019), thereby reducing oxidative stress and inflammation in patients with CKD (Zhang, et al. 2022). In detail, ginsenoside Rg1 treatment significantly reduced ROS production and inhibited NOX4 and NLRP3 inflammatory vesicle activation, which in turn ameliorated LPS-induced chronic kidney injury and renal fibrosis (Zhang et al. 2022). Rb1 can also inhibit the Wnt/β-linked protein pathway by activating peroxisome proliferator-activated receptor *γ* (PPAR-*γ*) to exert anti-calcium properties and thus improve the symptoms of CKD. In recent years, studies have found that ginsenoside Rb1 can regulate Type two diabetic nephropathy by regulating the expression of miR-3550 and further combining with Wnt/β-catenin signaling (Shao et al. 2019) or inhibiting aldose reductase activity (He et al. 2022b).

#### 3.1.2 Ginsenoside Rb3

Ginsenoside Rb3, which is one of the main pharmacologically active ingredients, mainly exists in the roots, flower buds, stems, and leaves of Panax ginseng; the roots, stems, and leaves of Panax quinquefolium; and the stems and leaves of Panax notoginseng. According to reports, ginsenoside Rb3 can regulate cisplatin-induced AKI by regulating AMPK/mTOR-mediated autophagy and inhibiting apoptosis *in vitro* and *in vivo* (Xing et al. 2019). Among them, Li et al. demonstrated for the first time the protective effect and potential mechanism of ginsenoside Rb3 on cisplatin-induced renal failure, restoring the antioxidant system by regulating the AMPK/mTOR signaling pathway, and inhibiting proximal tubular damage by inhibiting ROS-mediated apoptosis and autophagy. Some evidence suggests that the TGF-β pathway may lead to cisplatin-induced nephrotoxicity, and ginsenoside Rb3 can have a protective effect on nephrotoxicity in the treatment of oral cancer with CPT through TGF-β pathway-mediated mitochondrial apoptosis (Wu et al. 2021b).

#### 3.1.3 Ginsenoside Rd

Ginsenoside Rd is a rare type of saponin. The content of Rd in ginseng is very low, while that in Panax notoginseng is approximately 0.36%–1.47%, which is higher than that in ginseng. Intestinal enzymes can metabolize Rb1 with high content into Rd; therefore, Rd is one of the important forms of saponins that are absorbed and utilized by the intestine after metabolism. Recent studies have found that ginsenoside Rd has strong biological activity, especially protective effects on the kidneys. For example, ginsenosides-Rd eliminate the damaging effects of oxidative stress on the kidneys by inhibiting free radical lipid peroxidation (Yokozawa and Liu 2000). Moreover, ginsenoside Rd can regulate cisplatin-induced AKI by inhibiting free radical-mediated lipid peroxidation while inhibiting apoptosis (Yokozawa and Dong 2001). In the research of exploring new anti-kidney cancer drugs, ginsenoside Rd has played an important role. Studies have said that ginsenoside Rd can regulate kidney cancer by inhibiting TRPM7 channel activity (Kim 2013).

#### 3.1.4 Ginsenoside Rg3

Ginsenoside Rg3 is one of the main active substances in ginseng and has extensive pharmacological effects. With the new foci of the research, Rg3 was found to have anti-tumor effects (Sin et al. 2012; Ding et al. 2015), reducing the cardiotoxicity and nephrotoxicity of chemotherapy drugs (Han et al. 2016), and anti-cicatricial (Tang et al. 2018). Through literature reading, we find that there is a growing number of studies focusing on ginsenoside Rg3 to improve acute kidney injury. For example, Rg3 can regulate D-galactose-induced AKI by inhibiting the renal oxidative stress caused by d-galactose and simultaneously activating the PI3K/AKT signaling pathway to attenuate the apoptosis of liver and kidney cells (Sun, et al. 2013). Rg3 can regulate LPS-induced AKI by reducing the expression of NF-kB and iNOS proteins and reducing the expression of COX-2 and HO-1 proteins (Kang, et al. 2007). Numerous studies have also shown that ginsenoside Rg3 can improve cisplatin-induced AKI by modulating multiple pathways. In details, ginsenosides Rg3 can regulate cisplatin-induced AKI by regulating PI3K/AKT and NF-κB-mediated apoptosis and inflammatory pathways (Zhang et al. 2021). Ginsenosides Rg3 also reduces cisplatin-induced AKI by inhibiting apoptosis and autophagy to suppress NLRP3 (Zhai et al. 2021) and blocking the JNK-p53-cysteine asparticase-3 signaling cascade (Han et al. 2016). In addition, Rg3 can also regulate type 2 diabetic nephropathy mainly focusing on pathways that regulate MAPK/NF-κB signaling pathway (Li et al. 2021), inhibit inflammation and oxidative stress response (Zhou et al. 2020) and late glycosylation end product formation (Kang et al. 2010b). There have also been recent studies that Rg3 can regulate kidney cancer by blockading of TRPM7 channel activity (Kim et al. 2011).

#### 3.1.5 Ginsenoside Rh2

Ginsenoside Rh2 is a rare saponin found in Panax ginseng. Rh2 was first found in red ginseng and was later isolated from American ginseng, Panax notoginseng, and other plants. Rh2 can regulate the immune, central nervous, endocrine, and cardiovascular systems, etc., and has anti-tumor, anti-allergy, anti-depression, anti-aging, and improved myocardial ischemic effects. Ginsenoside Rh2 regulates cisplatin-induced AKI by acting on a caspase-mediated pathway (Qi, et al. 2019). Rh2 regulates type two diabetic nephropathy by downregulating discoid domain receptor 1 (Shen, et al. 2021).

#### 3.1.6 Ginsenoside CK(M1)

Ginsenoside CK does not exist in natural ginseng but is a metabolite produced by diol-type ginsenosides (such as Rb1, Rb2, and Rc) under the action of intestinal flora after oral administration (Chen, et al. 2015). CK is one of the main components of ginsenosides that play a role in the body with high biological activity, including inhibition of T Cells (Kang, et al. 2010a), promotion of tumor cell apoptosis (Cho, et al. 2009), anti-inflammation (Joh et al. 2011) and protection of the myocardium (Tsutsumi, et al. 2011). Ginsenoside CK can modulate primary glomerulonephritis by inhibiting NLRP3 inflammasome activation in renal tissue, macrophages, and bone marrow-derived dendritic cells, increasing SIRT1 expression, and triggering autophagy-mediated inhibition of NLRP3 inflammable bodies (Wu, et al. 2020). Ginsenoside CK can regulate tubulointerstitial nephritis by inhibiting NLRP3 inflammasome initiation and mitochondria-related activation signaling in tubulointerstitial lesions (Hsu, et al. 2020). Ginsenoside CK can regulate type two DN by inhibiting NLRP3 inflammasome activation and the NF-κB/p38 signaling pathway in DN in high-fat diet/streptozotocin-induced diabetic mice (Song, et al. 2018) enhancing antioxidant capacity, and reducing the damage of TGF-β1 in renal tissue (Shao, et al. 2015). Ginsenoside CK regulates kidney cancer by regulating reactive oxygen species (ROS) and Testis-associated highly conserved oncogenic long-stranded non-coding RNA (LNRNA THOR) (Chen, et al. 2021). Ginsenoside M1 can regulate acute severe lupus nephritis by inhibiting NLRP3 inflammasome activation and differentially regulating T-cell function (Lin, et al. 2019).

### 3.2 PPT type ginsenosides

PPT-type ginsenosides take protopanaxatriol as an aglycone. Generally, ginsenosides are divided into two configurations: 20 S)—PPT and 20 R)—PPT, according to the substitution of C-6 or C-20 hydroxyl groups of aglycones by different sugar groups. The structural formulae of common PPT-type ginsenosides are shown in Figure 1. The PPT ginsenoside group has been shown to have significant pharmacological activities, mainly Re, Rg1, Rg2, and Rh1, as shown in Table 3.

**TABLE 3:** Pharmacological activity of PPT type ginsenosides.

Ginsenoside	Activity	References
Re	Inhibits proliferation and protects nerves	Cai et al. (2022)
Rg1	Neuroprotective effect, induction of apoptosis, estrogen-like effect	Chan et al. (2002)
Rg2	Protection of central and peripheral nervous system	Sala et al. (2002)
Rh1	Boost immunity, estrogen-like, inhibit proliferation	Popovich and Kitts (2002)
		Lee et al. (2003b)

#### 3.2.1 Ginsenoside Rg1

Among all the PPT saponins, ginsenoside Rg1 ranks second only to ginsenoside Re. In addition, studies have shown that the ginsenoside Rg1 content in Panax notoginseng is high (Rg1 accounts for 20% of the total saponins of Panax notoginseng, and Re is only 2.5%), and thus has great development value (Yang, et al. 2015). Rg1 has many functions including anti-aging, anti-oxidation, and improved immunity and memory (monakhov, Baisong et al. 2018). Ginsenoside Rg1 can regulate D-galactose-induced AKI by preventing DNA damage by attenuating oxidative stress (Fan, et al. 2016). Rg1 can regulate CKD by inhibiting NOX4-NLRP3 inflammazone signaling pathways in mice (Liu, et al. 2010). Rg1 can regulate anti-GBM GN, a rare autoimmune disease, by increasing renal blood flow (Hattori, et al. 1991) and activating NRF2 signaling (Guo, et al. 2019). Ginsenoside Rg1 can inhibit the development of renal fibrosis by modulating various pathways, such as the downregulation of protein TGF-β1 expression (Xie, et al. 2008a), blocking TEMT by inhibiting the expression of TSP-1 (Xie et al. 2008b), thereby inhibiting the activation of TGF-β1 (Li, et al. 2015), inhibiting endoplasmic reticulum stress-induced apoptosis in rats after unilateral ureteral obstruction (Xie et al. 2008c), inhibiting TGF-β1-induced transdifferentiation of rat renal tubular epithelial cells (Shen, et al. 2020), inhibiting NOX4 and NLRP3 inflammasome activation in SAMP8 mice, and regulating the Klotho/TGF-β1/Smad signaling pathway (Li et al. 2018). Ginsenoside Rg1 can inhibit the development of type 2 DN by regulating the PI3K/AKT/FOXO3 pathway (Liu, et al. 2021), reducing the expression of TGF-β1 and the already mentioned inflammatory response factors in renal tissue (Ma et al. 2010), and reducing the expression of TNF-α and MCP-1 (Zhang, et al. 2009). The combination of Rg1 and Astragalus IV can reduce oxidative stress and inhibit the TGF-β1/Smad signaling cascade in renal fibrosis in rats with DN (Du, et al. 2018).

#### 3.2.2 Ginsenoside Rg2 and Rh1

Rg2 is an intermediate product of ginsenoside Re metabolism *in vivo*. Rg2 has many biological activities, such as affecting the sensitivity of the opposite nerve cell process receptors, promoting intercellular communication, and reducing the neural activity caused by electricity in the rat hippocampus (Sala, et al. 2002). Ginsenoside Rg2 and Rh1 can regulate AKI by blocking LPS-TLR4 signaling, which reduces p38-STAT1 activation and NF-κB translocation, which in turn suppresses the transcription of inflammatory cytokines and mediators, such as IFN-β, TNF-*α*, IL-1β, and iNOS (Huynh, et al. 2020).

#### 3.2.3 Ginsenoside Rh1

Ginsenoside Rh1 is a rare saponin found in red ginseng, Panax notoginseng, and American ginseng in trace (Jeon, et al. 2020). Because of its remarkable immunoregulatory activity, Rh1 has high medicinal value in the treatment of many senile diseases (Tam, et al. 2018). In addition, Rh1 can inhibit inflammatory reaction (Vinh et al. 2017), regulate abnormal immune responses in hypersensitive disease (Han and Kim, 2020), and inhibit tumor cell proliferation (Yi, 2019). Ginsenoside Rh1 can regulate type two DN by regulating oxidative stress, angiotensin II (Ang-II), and inflammatory processes, as well as AMPK/PI3K/Akt-mediated inflammatory and apoptosis signaling pathways (Su, et al. 2021).

### 3.3 Other ginsenosides

Other types of ginsenosides include dammarane-type tetracyclic triterpenoids. Some of these saponins are isolated from ginseng with very little content, whereas others are obtained after chemical treatment. Compared with the original PPD- and PPT-type ginsenosides, only the skeleton of aglycone or the side chain of the parent nucleus was partially changed.

#### 3.3.1 Ginsenoside Rg5

Ginsenoside Rg5 is a derivative of ginsenoside, one of the main components of red ginseng (Kim, et al. 1996). Rg5 is a secondary saponin obtained from PPD-type saponins (Rb1, Rb2, Rb3, Rc, and Rd) *via* regioselective hydrolysis and stereoselective dehydration (Lee, et al. 2009). In animal and human clinical trials, Rg5 not only has significant effects in improving lung inflammation (Kim et al. 2012) and improving memory (Yao et al. 2014), anti-cancer (Liang et al. 2015), but also reduces cisplatin induced nephrotoxicity (Li et al. 2016). Ginsenoside Rh3 can regulate cisplatin-induced AKI by inhibiting inflammation, oxidative stress, and apoptosis (Li et al. 2016). Ginsenoside Rh3 can inhibit the development of type two diabetic nephropathy by inhibiting NLRP3 inflammasome activation and the MAPK signaling pathway in high-fat diet/streptozotocin-induced diabetic mice (Zhu, et al. 2020).

#### 3.3.2 Ginsenoside Rh3

Ginsenoside Rh3 is a metabolite of ginsenoside Rg5 in the human body (Kim et al. 2013), but it has better effects on various pharmacological activities than Rg5 (Shin et al. 2006). Ginsenoside Rh3 can regulate cisplatin-induced AKI by inhibiting JNK and ERK mitogen-activated protein kinase signaling cascades (Lee and Kang, 2017).

#### 3.3.3 Ginsenoside Rg3, Rg5 and Rk1

Black ginseng, a new processed product of ginseng, has a unique processing method (Gao, et al. 2017). Black ginseng contains rare saponins that are different from ginseng and red ginseng, and its representative active components are Rg3, Rg5, and Rk1 (Gao, et al. 2017). As a tetracyclic triterpene discovered in recent years, Rk1 has attracted much attention because of its biological activities, such as antitumor, blood glucose regulation, and nervous system protection (Gao, et al. 2017). Ginsenoside Rk1 regulates cisplatin-induced AKI by regulating inflammation and apoptosis (Park, et al. 2015).

#### 3.3.4 Ginsenoside Rh4 and Rk3

Ginsenoside Rk3/Rh4 comprises a pair of isomers, which are obtained by removing a sugar group from ginsenoside Rg1 and converting it to Rh1, and then removing a water molecule at C20 position from Rh1. Ginsenoside Rh4 and Rk3 can regulate cisplatin-induced AKI by inhibiting oxidation (Baek, et al. 2006).

## 4 Prospect and conclusion

Nowadays, kidney-related diseases have become common worldwide, such as diabetic nephropathy, chronic kidney disease, acute kidney disease, and hypertensive nephropathy. The causes of kidney diseases are complex, and since the pathogenesis of most kidney diseases is not fully understood, the treatment of kidney diseases is mostly empirical and lacks etiologic treatment tools (Liu and Xiao 1992). At the same time, many drugs that protect the kidney have been widely used, but some of them have strong adverse effects. For this reason, monomeric components derived from traditional Chinese herbal medicines have received much attention as effective and safe alternative drugs for kidney diseases. Currently, ginsenosides as natural drugs have been widely approved to exert therapeutic effects on kidney-related drugs *in vivo* and *in vitro*. In the existing studies, we found that ginsenosides for the treatment of nephropathy mainly focus on mechanisms through the inhibition of inflammatory responses and oxidative stress. Emerging reports suggest that ginsenosides can have some binding activity to the glucocorticoid receptor and can promote its nuclear translocation, while some ginsenosides can exert anti-inflammatory effects by inhibiting the activity of NF-κB. Recent findings have confirmed that NF-κB is closely associated with foot cell injury. This shows that NF-κB pathway is a hot pathway for kidney disease research. Current research mainly focuses on ginsenosides, with little research on other components such as ginsenosides polysaccharides, which should be more widely explored in depth. The efficacy of ginsenosides in the treatment of kidney disease has become clear, and in the future, in-depth research on ginsenosides in the treatment of kidney disease can be conducted from multiple angles, levels and directions to provide better treatment for patients.

In conclusion, the pathogenesis of several major kidney-related diseases is discussed in this review. The mechanism of ginsenosides in various types of nephropathy has been summarized, including PPD and PPT types. Although ginsenosides have been studied in nephropathy, their mechanism of action has not been fully elucidated. The animal model of nephropathy was used as the basis for further research and discussion of the pathogenesis and mechanism of drug action. The establishment of animal models of nephropathy should be consistent with the pathology and course of kidney disease, which is a goal of researchers. Therefore, it is necessary to further study other related effects of ginsenosides on kidney-related diseases through appropriate animal models, which are expected to develop new drugs for the clinical treatment of drug-induced nephrotoxic diseases, diabetic nephropathy, renal fibrosis, and other kidney diseases.

## References

[B1] Anders H. J. Fogo A. B. (2014). Immunopathology of lupus nephritis. Semin Immunopathol 36 (4), 443–459. 10.1007/s00281-013-0413-5 24402709

[B2] Bae E. A. Han M. J. Kim E. J. Kim D. H. (2004). Transformation of ginseng saponins to ginsenoside Rh2 by acids and human intestinal bacteria and biological activities of their transformants. Arch Pharm Res 27 (1), 61–67. 10.1007/BF02980048 14969341

[B3] Baek S. H. Piao X. L. Lee U. J. Kim H. Y. Park J. H. (2006). Reduction of Cisplatin-induced nephrotoxicity by ginsenosides isolated from processed ginseng in cultured renal tubular cells. Biol Pharm Bull 29 (10), 2051–2055. 10.1248/bpb.29.2051 17015950

[B4] Baisong Z. [j] K. m. Yameng L. li P.-y. (2018). Progress in bioactivity of Ginsenoside Rg1 %J. Chinese Journal of Traditional Chinese Medicine 33 (04), 1463–1465.

[B5] Berek L. Szabó D. Petri I. B. Shoyama Y. Lin Y. H. Molnár J. (2001). Effects of naturally occurring glucosides, solasodine glucosides, ginsenosides and parishin derivatives on multidrug resistance of lymphoma cells and leukocyte functions. Vivo 15 (2), 151–156. 11317520

[B6] Bucaloiu I. D. Kirchner H. L. Norfolk E. R. Hartle J. E. 2nd Perkins R. M. (2012). Increased risk of death and de novo chronic kidney disease following reversible acute kidney injury. Kidney Int 81 (5), 477–485. 10.1038/ki.2011.405 22157656

[B7] Burns W. C. Twigg S. M. Forbes J. M. Pete J. Tikellis C. Thallas-Bonke V. Thomas M. C. Cooper M. E. Kantharidis P. (2006). Connective tissue growth factor plays an important role in advanced glycation end product-induced tubular epithelial-to-mesenchymal transition: implications for diabetic renal disease. J Am Soc Nephrol 17 (9), 2484–2494. 10.1681/ASN.2006050525 16914537

[B8] Cai J. Huang K. Han S. Chen R. Li Z. Chen Y. Chen B. Li S. Xinhua L. Yao H. (2022). A comprehensive system review of pharmacological effects and relative mechanisms of Ginsenoside Re: Recent advances and future perspectives. Phytomedicine 102, 154119. 10.1016/j.phymed.2022.154119 35617888

[B9] Calvier L. Miana M. Reboul P. Cachofeiro V. Martinez-Martinez E. de Boer R. A. Poirier F. Lacolley P. Zannad F. Rossignol P. López-Andrés N. (2013). Galectin-3 mediates aldosterone-induced vascular fibrosis. Arterioscler Thromb Vasc Biol 33 (1), 67–75. 10.1161/ATVBAHA.112.300569 23117656

[B10] Chan R. Y. Chen W. F. Dong A. Guo D. Wong M. S. (2002). Estrogen-like activity of ginsenoside Rg1 derived from Panax notoginseng. J Clin Endocrinol Metab 87 (8), 3691–3695. 10.1210/jcem.87.8.8717 12161497

[B11] Chawla L. S. Kimmel P. L. (2012). Acute kidney injury and chronic kidney disease: an integrated clinical syndrome. Kidney Int 82 (5), 516–524. 10.1038/ki.2012.208 22673882

[B12] Chawla L. S. Eggers P. W. Star R. A. Kimmel P. L. (2014). Acute kidney injury and chronic kidney disease as interconnected syndromes. N Engl J Med 371 (1), 58–66. 10.1056/NEJMra1214243 24988558 PMC9720902

[B13] Chen ling guo J. p. (2019). Research progress of Radix sophora nigricans. %J Ginseng study 31 (01), 42–46.

[B14] Chen J. Wu H. Wang Q. Chang Y. Liu K. Wei W. (2015). Ginsenoside metabolite compound K suppresses T-cell priming via modulation of dendritic cell trafficking and costimulatory signals, resulting in alleviation of collagen-induced arthritis. J Pharmacol Exp Ther 353 (1), 71–79. 10.1124/jpet.114.220665 25630466

[B15] Chen Y. Chen J. Jiang M. Fu Y. Zhu Y. Jiao N. Liu L. Du Q. Wu H. Xu H. Sun J. (2020). Loganin and catalpol exert cooperative ameliorating effects on podocyte apoptosis upon diabetic nephropathy by targeting AGEs-RAGE signaling. Life Sci 252, 117653. 10.1016/j.lfs.2020.117653 32277978

[B16] Chen S. Ye H. Gong F. Mao S. Li C. Xu B. Ren Y. Yu R. (2021). Ginsenoside compound K exerts antitumour effects in renal cell carcinoma via regulation of ROS and lncRNA THOR. Oncol Rep 45 (4), 38. 10.3892/or.2021.7989 33649829 PMC7905530

[B17] Cho S. H. Chung K. S. Choi J. H. Kim D. H. Lee K. T. (2009). Compound K, a metabolite of ginseng saponin, induces apoptosis via caspase-8-dependent pathway in HL-60 human leukemia cells. BMC Cancer 9, 449. 10.1186/1471-2407-9-449 20017956 PMC2806409

[B18] Coresh J. Astor B. C. Greene T. Eknoyan G. Levey A. S. (2003). Prevalence of chronic kidney disease and decreased kidney function in the adult US population: Third National Health and Nutrition Examination Survey. Am J Kidney Dis 41 (1), 1–12. 10.1053/ajkd.2003.50007 12500213

[B19] Dieker J. W. Fransen J. H. van Bavel C. C. Briand J. P. Jacobs C. W. Muller S. Berden J. H. van der Vlag J. (2007). Apoptosis-induced acetylation of histones is pathogenic in systemic lupus erythematosus. Arthritis Rheum 56 (6), 1921–1933. 10.1002/art.22646 17530637

[B20] Ding S. Li C. Cheng N. Cui X. Xu X. Zhou G. (2015). Redox Regulation in Cancer Stem Cells. Oxid Med Cell Longev 2015, 750798. 10.1155/2015/750798 26273424 PMC4529979

[B21] Diwan V. Brown L. Gobe G. C. (2018). Adenine-induced chronic kidney disease in rats. Nephrology (Carlton) 23 (1), 5–11. 10.1111/nep.13180 29030945

[B22] Du N. Xu Z. Gao M. Liu P. Sun B. Cao X. (2018). Combination of Ginsenoside Rg1 and Astragaloside IV reduces oxidative stress and inhibits TGF-β1/Smads signaling cascade on renal fibrosis in rats with diabetic nephropathy. Drug Des Devel Ther 12, 3517–3524. 10.2147/DDDT.S171286 PMC6201993 30425453

[B23] Fan Y. Xia J. Jia D. Zhang M. Zhang Y. Huang G. Wang Y. (2016). Mechanism of ginsenoside Rg1 renal protection in a mouse model of d-galactose-induced subacute damage. Pharm Biol 54 (9), 1815–1821. 10.3109/13880209.2015.1129543 26730750

[B24] Feng F. Nyland J. Banyai M. Tatum A. Silverstone A. E. Gavalchin J. (2010). The induction of the lupus phenotype by estrogen is via an estrogen receptor-alpha-dependent pathway. Clin Immunol 134 (2), 226–236. 10.1016/j.clim.2009.10.004 19926524

[B25] Fernandes R. Freitas S. Cunha P. Alves G. Cotter J. (2016). Goodpasture's syndrome with absence of circulating anti-glomerular basement membrane antibodies: a case report. J Med Case Rep 10, 205. 10.1186/s13256-016-0984-6 27459964 PMC4962374

[B26] Fujimoto J. Sakaguchi H. Aoki I. Toyoki H. Khatun S. Tamaya T. (2001). Inhibitory effect of ginsenoside-Rb2 on invasiveness of uterine endometrial cancer cells to the basement membrane. Eur J Gynaecol Oncol 22 (5), 339–341. 11766734

[B27] Gao Y. Chu S. Zhang Z. Chen N. (2017). Hepataprotective effects of ginsenoside Rg1 - A review. J Ethnopharmacol 206, 178–183. 10.1016/j.jep.2017.04.012 28427912

[B28] Gentle M. E. Shi S. Daehn I. Zhang T. Qi H. Yu L. D'Agati V. D. Schlondorff D. O. Bottinger E. P. (2013). Epithelial cell TGFβ signaling induces acute tubular injury and interstitial inflammation. J Am Soc Nephrol 24 (5), 787–799. 10.1681/ASN.2012101024 23539761 PMC3636798

[B29] Gewin L. Zent R. Pozzi A. (2017). Progression of chronic kidney disease: too much cellular talk causes damage. Kidney Int 91 (3), 552–560. 10.1016/j.kint.2016.08.025 27773427 PMC5313325

[B30] Guo X. Zhang J. Liu M. Zhao G. C. (2019). Protective effect of ginsenoside Rg1 on attenuating anti-GBM glomerular nephritis by activating NRF2 signalling. Artif Cells Nanomed Biotechnol 47 (1), 2972–2979. 10.1080/21691401.2019.1640712 31322005

[B31] Guo M. Shao S. Wang D. Zhao D. Wang M. (2021). Recent progress in polysaccharides from Panax ginseng C A. Meyer. Food Funct 12 (2), 494–518. 10.1039/d0fo01896a 33331377

[B32] Guo J. Wang R. Min F. (2022). Ginsenoside Rg1 ameliorates sepsis-induced acute kidney injury by inhibiting ferroptosis in renal tubular epithelial cells. J Leukoc Biol 112, 1065–1077. 10.1002/JLB.1A0422-211R 35774015

[B33] Han M. J. Kim D. H. (2020). Effects of Red and Fermented Ginseng and Ginsenosides on Allergic Disorders. Biomolecules 10 (4), 634. 10.3390/biom10040634 32326081 PMC7226199

[B34] Han M. S. Han I. H. Lee D. An J. M. Kim S. N. Shin M. S. Yamabe N. Hwang G. S. Yoo H. H. Choi S. J. Kang K. S. Jang H. J. (2016). Beneficial effects of fermented black ginseng and its ginsenoside 20(S)-Rg3 against cisplatin-induced nephrotoxicity in LLC-PK1 cells. J Ginseng Res 40 (2), 135–140. 10.1016/j.jgr.2015.06.006 27158234 PMC4845053

[B35] Hattori T. Ito M. Suzuki Y. (1991). Studies on antinephritic effects of plant components in rats (2): Effects of ginsenosides on original-type anti-GBM nephritis in rats and its mechanisms. Nihon Yakurigaku Zasshi 97 (2), 127–134. 10.1254/fpj.97.2_127 1829061

[B36] He B. Chen D. Zhang X. Yang R. Yang Y. Chen P. Shen Z. (2022a). Oxidative Stress and Ginsenosides: An Update on the Molecular Mechanisms. Oxid Med Cell Longev 2022, 9299574. 10.1155/2022/9299574 35498130 PMC9045968

[B37] He J. Y. Hong Q. Chen B. X. Cui S. Y. Liu R. Cai G. Y. Guo J. Chen X. M. (2022b). Ginsenoside Rb1 alleviates diabetic kidney podocyte injury by inhibiting aldose reductase activity. Acta Pharmacol Sin 43 (2), 342–353. 10.1038/s41401-021-00788-0 34811512 PMC8791932

[B38] Hristova M. H. Stoyanova V. S. (2017). Autoantibodies against complement components in systemic lupus erythematosus - role in the pathogenesis and clinical manifestations. Lupus 26 (14), 1550–1555. 10.1177/0961203317709347 29092674

[B39] Hsu W. H. Hua K. F. Tuan L. H. Tsai Y. L. Chu L. J. Lee Y. C. Wong W. T. Lee S. L. Lai J. H. Chu C. L. Ho L. J. Chiu H. W. Hsu Y. J. Chen C. H. Ka S. M. Chen A. (2020). Compound K inhibits priming and mitochondria-associated activating signals of NLRP3 inflammasome in renal tubulointerstitial lesions. Nephrol Dial Transplant 35 (1), 74–85. 10.1093/ndt/gfz073 31065699

[B40] Humphreys B. D. (2018). Mechanisms of Renal Fibrosis. Annu Rev Physiol 80, 309–326. 10.1146/annurev-physiol-022516-034227 29068765

[B41] Huynh D. T. N. Baek N. Sim S. Myung C. S. Heo K. S. (2020). Minor Ginsenoside Rg2 and Rh1 Attenuates LPS-Induced Acute Liver and Kidney Damages via Downregulating Activation of TLR4-STAT1 and Inflammatory Cytokine Production in Macrophages. Int J Mol Sci 21 (18), 6656. 10.3390/ijms21186656 32932915 PMC7555743

[B42] Jeon J. H. Lee S. Lee W. Jin S. Kwon M. Shin C. H. Choi M. K. Song I. S. (2020). Herb-Drug Interaction of Red Ginseng Extract and Ginsenoside Rc with Valsartan in Rats. Molecules 25 (3), 622. 10.3390/molecules25030622 32023909 PMC7037682

[B43] Joh E. H. Lee I. A. Jung I. H. Kim D. H. (2011). Ginsenoside Rb1 and its metabolite compound K inhibit IRAK-1 activation--the key step of inflammation. Biochem Pharmacol 82 (3), 278–286. 10.1016/j.bcp.2011.05.003 21600888

[B44] Kang K. S. Kim H. Y. Yamabe N. Park J. H. Yokozawa T. (2007). Preventive effect of 20(S)-ginsenoside Rg3 against lipopolysaccharide-induced hepatic and renal injury in rats. Free Radic Res 41 (10), 1181–1188. 10.1080/10715760701581740 17886040

[B45] Kang K. S. Yamabe N. Kim H. Y. Park J. H. Yokozawa T. (2010a). Effects of heat-processed ginseng and its active component ginsenoside 20(S)-Rg3 on the progression of renal damage and dysfunction in type 2 diabetic Otsuka Long-Evans Tokushima Fatty rats. Biol Pharm Bull 33 (6), 1077–1081. 10.1248/bpb.33.1077 20522983

[B46] Kang X. Chen J. Qin Q. Wang F. Wang Y. Lan T. Xu S. Wang F. Xia J. Ekberg H. Qi Z. Liu Z. (2010b). Isatis tinctoria L. combined with co-stimulatory molecules blockade prolongs survival of cardiac allografts in alloantigen-primed mice. Transpl Immunol 23 (1-2), 34–39. 10.1016/j.trim.2010.03.006 20338239

[B47] Ke B. Zhang A. Wu X. Fang X. (2015). The Role of Krüppel-like Factor 4 in Renal Fibrosis. Front Physiol 6, 327. 10.3389/fphys.2015.00327 26617530 PMC4641914

[B48] Keum Y. S. Han S. S. Chun K. S. Park K. K. Park J. H. Lee S. K. Surh Y. J. (2003). Inhibitory effects of the ginsenoside Rg3 on phorbol ester-induced cyclooxygenase-2 expression, NF-kappaB activation and tumor promotion. Mutat Res 523-524, 75–85. 10.1016/s0027-5107(02)00323-8 12628505

[B49] Kim Y. S. Jin S. H. (2004). Ginsenoside Rh2 induces apoptosis via activation of caspase-1 and -3 and up-regulation of Bax in human neuroblastoma. Arch Pharm Res 27 (8), 834–839. 10.1007/BF02980175 15460444

[B50] Kim S. I. Park J. H. Ryu J.-H. Park J. D. Lee Y. H. Park J.-H. Kim T.-H. Kim J. M. Baek N.-I. (1996). Ginsenoside Rg5, a genuine dammarane glycoside from Korean red ginseng. Archives of Pharmacal Research 19 (6), 551–553. 10.1007/bf02986026 18975165

[B51] Kim B. J. Nah S. Y. Jeon J. H. So I. Kim S. J. (2011). Transient receptor potential melastatin 7 channels are involved in ginsenoside Rg3-induced apoptosis in gastric cancer cells. Basic Clin Pharmacol Toxicol 109 (4), 233–239. 10.1111/j.1742-7843.2011.00706.x 21443732

[B52] Kim T. W. Joh E. H. Kim B. Kim D. H. (2012). Ginsenoside Rg5 ameliorates lung inflammation in mice by inhibiting the binding of LPS to toll-like receptor-4 on macrophages. Int Immunopharmacol 12 (1), 110–116. 10.1016/j.intimp.2011.10.023 22107725

[B53] Kim E. J. Jung I. H. Van Le T. K. Jeong J. J. Kim N. J. Kim D. H. (2013). Ginsenosides Rg5 and Rh3 protect scopolamine-induced memory deficits in mice. J Ethnopharmacol 146 (1), 294–299. 10.1016/j.jep.2012.12.047 23313392

[B54] Kim B. J. (2013). Involvement of melastatin type transient receptor potential 7 channels in ginsenoside Rd-induced apoptosis in gastric and breast cancer cells. J Ginseng Res 37 (2), 201–209. 10.5142/jgr.2013.37.201 23717173 PMC3659640

[B55] Lahmer T. Heemann U. (2012). Anti-glomerular basement membrane antibody disease: a rare autoimmune disorder affecting the kidney and the lung. Autoimmun Rev 12 (2), 169–173. 10.1016/j.autrev.2012.04.002 22546293

[B56] Lee H. L. Kang K. S. (2017). Protective effect of ginsenoside Rh3 against anticancer drug-induced apoptosis in LLC-PK1 kidney cells. J Ginseng Res 41 (2), 227–231. 10.1016/j.jgr.2017.01.011 28413329 PMC5386128

[B57] Lee B. H. Lee S. J. Hur J. H. Lee S. Sung J. H. Huh J. D. Moon C. K. (1998). *In vitro* antigenotoxic activity of novel ginseng saponin metabolites formed by intestinal bacteria. Planta Med 64 (6), 500–503. 10.1055/s-2006-957501 9741293

[B58] Lee Y. J. Jin Y. R. Lim W. C. Park W. K. Cho J. Y. Jang S. Lee S. K. (2003a). Ginsenoside-Rb1 acts as a weak phytoestrogen in MCF-7 human breast cancer cells. Arch Pharm Res 26 (1), 58–63. 10.1007/BF03179933 12568360

[B59] Lee Y. Jin Y. Lim W. Ji S. Choi S. Jang S. Lee S. (2003b). A ginsenoside-Rh1, a component of ginseng saponin, activates estrogen receptor in human breast carcinoma MCF-7 cells. J Steroid Biochem Mol Biol 84 (4), 463–468. 10.1016/s0960-0760(03)00067-0 12732291

[B60] Lee J. Y. Shin J. W. Chun K. S. Park K. K. Chung W. Y. Bang Y. J. Sung J. H. Surh Y. J. (2005). Antitumor promotional effects of a novel intestinal bacterial metabolite (IH-901) derived from the protopanaxadiol-type ginsenosides in mouse skin. Carcinogenesis 26 (2), 359–367. 10.1093/carcin/bgh313 15498788

[B61] Lee S. M. Shon H. J. Choi C. S. Hung T. M. Min B. S. Bae K. (2009). Ginsenosides from heat processed ginseng. Chem Pharm Bull (Tokyo) 57 (1), 92–94. 10.1248/cpb.57.92 19122325

[B62] Li S. S. Ye J. M. Deng Z. Y. Yu L. X. Gu X. X. Liu Q. F. (2015). Ginsenoside-Rg1 inhibits endoplasmic reticulum stress-induced apoptosis after unilateral ureteral obstruction in rats. Ren Fail 37 (5), 890–895. 10.3109/0886022X.2015.1015427 25707520

[B63] Li W. Yan M. H. Liu Y. Liu Z. Wang Z. Chen C. Zhang J. Sun Y. S. (2016). Ginsenoside Rg5 Ameliorates Cisplatin-Induced Nephrotoxicity in Mice through Inhibition of Inflammation, Oxidative Stress, and Apoptosis. Nutrients 8 (9), 566. 10.3390/nu8090566 27649238 PMC5037551

[B64] Li S. S. He A. L. Deng Z. Y. Liu Q. F. (2018). Ginsenoside-Rg1 Protects against Renal Fibrosis by Regulating the Klotho/TGF-β1/Smad Signaling Pathway in Rats with Obstructive Nephropathy. Biol Pharm Bull 41 (4), 585–591. 10.1248/bpb.b17-00934 29607931

[B65] Li Y. Hou J. G. Liu Z. Gong X. J. Hu J. N. Wang Y. P. Liu W. C. Lin X. H. Wang Z. Li W. (2021). Alleviative effects of 20(R)-Rg3 on HFD/STZ-induced diabetic nephropathy via MAPK/NF-κB signaling pathways in C57BL/6 mice. J Ethnopharmacol 267, 113500. 10.1016/j.jep.2020.113500 33091499

[B66] Liang L. D. He T. Du T. W. Fan Y. G. Chen D. S. Wang Y. (2015). Ginsenoside-Rg5 induces apoptosis and DNA damage in human cervical cancer cells. Mol Med Rep 11 (2), 940–946. 10.3892/mmr.2014.2821 25355274 PMC4262516

[B67] Lin T. J. Wu C. Y. Tsai P. Y. Hsu W. H. Hua K. F. Chu C. L. Lee Y. C. Chen A. Lee S. L. Lin Y. J. Hsieh C. Y. Yang S. R. Liu F. C. Ka S. M. (2019). Accelerated and Severe Lupus Nephritis Benefits From M1, an Active Metabolite of Ginsenoside, by Regulating NLRP3 Inflammasome and T Cell Functions in Mice. Front Immunol 10, 1951. 10.3389/fimmu.2019.01951 31475012 PMC6702666

[B68] Liu C. X. Xiao P. G. (1992). Recent advances on ginseng research in China. J Ethnopharmacol 36 (1), 27–38. 10.1016/0378-8741(92)90057-x 1501490

[B69] Liu Z. Q. Luo X. Y. Sun Y. X. Chen Y. P. Wang Z. C. (2002). Can ginsenosides protect human erythrocytes against free-radical-induced hemolysis? Biochim Biophys Acta 1572 (1), 58–66. 10.1016/s0304-4165(02)00281-7 12204333

[B70] Liu Z. Q. Luo X. Y. Liu G. Z. Chen Y. P. Wang Z. C. Sun Y. X. (2003). *In vitro* study of the relationship between the structure of ginsenoside and its antioxidative or prooxidative activity in free radical induced hemolysis of human erythrocytes. J Agric Food Chem 51 (9), 2555–2558. 10.1021/jf026228i 12696936

[B71] Liu W. J. Tang H. T. Jia Y. T. Ma B. Fu J. F. Wang Y. Lv K. Y. Xia Z. F. (2010). Notoginsenoside R1 attenuates renal ischemia-reperfusion injury in rats. Shock 34 (3), 314–320. 10.1097/SHK.0b013e3181ceede4 20023602

[B72] Liu Z. Li C. Zhang Q. Tao M. (2012). Effect of Renshen polysaccharides on oxidative injury in kidney IR rabbits. Carbohydr Polym 90 (2), 773–777. 10.1016/j.carbpol.2012.05.040 22840000

[B73] Liu H. Lu X. Hu Y. Fan X. (2020). Chemical constituents of Panax ginseng and Panax notoginseng explain why they differ in therapeutic efficacy. Pharmacol Res 161, 105263. 10.1016/j.phrs.2020.105263 33127555

[B74] Liu H. Chen W. Lu P. Ma Y. Liang X. Liu Y. (2021). Ginsenoside Rg1 attenuates the inflammation and oxidative stress induced by diabetic nephropathy through regulating the PI3K/AKT/FOXO3 pathway. Ann Transl Med 9 (24), 1789. 10.21037/atm-21-6234 35071483 PMC8756242

[B75] Liu T. Zhu L. Wang L. (2022). A narrative review of the pharmacology of ginsenoside compound K. Ann Transl Med 10 (4), 234. 10.21037/atm-22-501 35280413 PMC8908159

[B76] Liu Y. (2006). Renal fibrosis: new insights into the pathogenesis and therapeutics. Kidney Int 69 (2), 213–217. 10.1038/sj.ki.5000054 16408108

[B77] Liu Y. (2010). New insights into epithelial-mesenchymal transition in kidney fibrosis. J Am Soc Nephrol 21 (2), 212–222. 10.1681/ASN.2008121226 20019167 PMC4554339

[B78] Lo L. J. Go A. S. Chertow G. M. McCulloch C. E. Fan D. Ordoñez J. D. Hsu C. Y. (2009). Dialysis-requiring acute renal failure increases the risk of progressive chronic kidney disease. Kidney Int 76 (8), 893–899. 10.1038/ki.2009.289 19641480 PMC2771754

[B79] López-Farré A. Gómez-Garre D. Bernabeu F. López-Novoa J. M. Lopez-FArre A. (1991). A role for endothelin in the maintenance of post-ischaemic renal failure in the rat. J Physiol 444, 513–522. 10.1113/jphysiol.1991.sp018891 1822562 PMC1179946

[B80] Ma X. Xie X. Zuo C. Fan J. (2010). Effects of ginsenoside Rg1 on streptozocin-induced diabetic nephropathy in rats. Sheng Wu Yi Xue Gong Cheng Xue Za Zhi 27 (2), 342–347. 20481316

[B81] Mehta R. L. Cerdá J. Burdmann E. A. Tonelli M. García-García G. Jha V. Susantitaphong P. Rocco M. Vanholder R. Sever M. S. Cruz D. Jaber B. Lameire N. H. Lombardi R. Lewington A. Feehally J. Finkelstein F. Levin N. Pannu N. Thomas B. Aronoff-Spencer E. Remuzzi G. (2015). International Society of Nephrology's 0by25 initiative for acute kidney injury (zero preventable deaths by 2025): a human rights case for nephrology. Lancet 385 (9987), 2616–2643. 10.1016/S0140-6736(15)60126-X 25777661

[B82] Meng H. Liu X. K. Li J. R. Bao T. Y. Yi F. (2022). Bibliometric analysis of the effects of ginseng on skin. J Cosmet Dermatol 21 (1), 99–107. 10.1111/jocd.14450 34520601

[B83] Miller R. P. Tadagavadi R. K. Ramesh G. Reeves W. B. (2010). Mechanisms of Cisplatin nephrotoxicity. Toxins (Basel) 2 (11), 2490–2518. 10.3390/toxins2112490 22069563 PMC3153174

[B84] Nangaku M. Rosenberger C. Heyman S. N. Eckardt K. U. (2013). Regulation of hypoxia-inducible factor in kidney disease. Clin Exp Pharmacol Physiol 40 (2), 148–157. 10.1111/1440-1681.12005 22905709

[B85] Natesan V. Kim S. J. (2021). Diabetic Nephropathy - a Review of Risk Factors, Progression, Mechanism, and Dietary Management. Biomol Ther (Seoul) 29 (4), 365–372. 10.4062/biomolther.2020.204 33888647 PMC8255138

[B86] Ni Y. H. Deng H. F. Zhou L. Huang C. S. Wang N. N. Yue L. X. Li G. F. Yu H. J. Zhou W. Gao Y. (2022). Ginsenoside Rb1 Ameliorated Bavachin-Induced Renal Fibrosis via Suppressing Bip/eIF2α/CHOP Signaling-Mediated EMT. Front Pharmacol 13, 872474. 10.3389/fphar.2022.872474 35873571 PMC9304982

[B87] Ostendorf T. Boor P. van Roeyen C. R. Floege J. (2014). Platelet-derived growth factors (PDGFs) in glomerular and tubulointerstitial fibrosis. Kidney Int Suppl 4 (1), 65–69. 10.1038/kisup.2014.12 PMC4536969 26312152

[B88] Park E. K. Shin Y. W. Lee H. U. Kim S. S. Lee Y. C. Lee B. Y. Kim D. H. (2005). Inhibitory effect of ginsenoside Rb1 and compound K on NO and prostaglandin E2 biosyntheses of RAW264.7 cells induced by lipopolysaccharide. Biol Pharm Bull 28 (4), 652–656. 10.1248/bpb.28.652 15802804

[B89] Park J. Y. Choi P. Kim T. Ko H. Kim H. K. Kang K. S. Ham J. (2015). Protective Effects of Processed Ginseng and Its Active Ginsenosides on Cisplatin-Induced Nephrotoxicity: *In Vitro* and *in Vivo* Studies. J Agric Food Chem 63 (25), 5964–5969. 10.1021/acs.jafc.5b00782 26050847

[B90] Popovich D. G. Kitts D. D. (2002). Structure-function relationship exists for ginsenosides in reducing cell proliferation and inducing apoptosis in the human leukemia (THP-1) cell line. Arch Biochem Biophys 406 (1), 1–8. 10.1016/s0003-9861(02)00398-3 12234484

[B91] Qi Z. Li W. Tan J. Wang C. Lin H. Zhou B. Liu J. Li P. (2019). Effect of ginsenoside Rh(2) on renal apoptosis in cisplatin-induced nephrotoxicity *in vivo* . Phytomedicine 61, 152862. 10.1016/j.phymed.2019.152862 31048124

[B92] Qu C. Bai Y. Jin X. Wang Y. Zhang K. You J. Zhang H. (2009). Study on ginsenosides in different parts and ages of Panax quinquefolius L. Food Chemistry 115 (1), 340–346. 10.1016/j.foodchem.2008.11.079

[B93] Reutens A. T. Atkins R. C. (2011). Epidemiology of diabetic nephropathy. Contrib Nephrol 170, 1–7. 10.1159/000324934 21659752

[B94] Romagnani P. Remuzzi G. Glassock R. Levin A. Jager K. J. Tonelli M. Massy Z. Wanner C. Anders H. J. (2017). Chronic kidney disease. Nat Rev Dis Primers 3, 17088. 10.1038/nrdp.2017.88 29168475

[B95] Sagoo M. K. Gnudi L. (2018). Diabetic nephropathy: Is there a role for oxidative stress? Free Radic Biol Med 116, 50–63. 10.1016/j.freeradbiomed.2017.12.040 29305106

[B96] Sala F. Mulet J. Choi S. Jung S. Y. Nah S. Y. Rhim H. Valor L. M. Criado M. Sala S. (2002). Effects of ginsenoside Rg2 on human neuronal nicotinic acetylcholine receptors. J Pharmacol Exp Ther 301 (3), 1052–1059. 10.1124/jpet.301.3.1052 12023537

[B97] Shao X. Li N. Zhan J. Sun H. An L. Du P. (2015). Protective effect of compound K on diabetic rats. Nat Prod Commun 10 (2), 1934578X1501000–245. 10.1177/1934578x1501000206 25920251

[B98] Shao X. Chen C. Miao C. Yu X. Li X. Geng J. Fan D. Lin X. Chen Z. Shi Y. (2019). Expression analysis of microRNAs and their target genes during experimental diabetic renal lesions in rats administered with ginsenoside Rb1 and trigonelline. Pharmazie 74 (8), 492–498. 10.1691/ph.2019.8903 31526443

[B99] Shen X. Dong X. Han Y. Li Y. Ding S. Zhang H. Sun Z. Yin Y. Li W. Li W. (2020). Ginsenoside Rg1 ameliorates glomerular fibrosis during kidney aging by inhibiting NOX4 and NLRP3 inflammasome activation in SAMP8 mice. Int Immunopharmacol 82, 106339. 10.1016/j.intimp.2020.106339 32114413

[B100] Shen B. Wang F. Zhou Y. Li T. He C. Zhao W. (2021). Ginsenoside Rh2 inhibits renal fibrosis and renal cell apoptosis in rats with diabetic nephropathy by downregulating discoid domain receptor 1. Nan Fang Yi Ke Da Xue Xue Bao 41 (7), 1107–1113. 10.12122/j.issn.1673-4254.2021.07.21 34308864 PMC8329676

[B101] Shin K. C. Oh D. K. (2016). Classification of glycosidases that hydrolyze the specific positions and types of sugar moieties in ginsenosides. Crit Rev Biotechnol 36 (6), 1036–1049. 10.3109/07388551.2015.1083942 26383974

[B102] Shin Y. W. Bae E. A. Kim D. H. (2006). Inhibitory effect of ginsenoside Rg5 and its metabolite ginsenoside Rh3 in an oxazolone-induced mouse chronic dermatitis model. Arch Pharm Res 29 (8), 685–690. 10.1007/BF02968253 16964764

[B103] Shin B. K. Kwon S. W. Park J. H. (2015). Chemical diversity of ginseng saponins from Panax ginseng. J Ginseng Res 39 (4), 287–298. 10.1016/j.jgr.2014.12.005 26869820 PMC4593792

[B104] Sin S. Kim S. Y. Kim S. S. (2012). Chronic treatment with ginsenoside Rg3 induces Akt-dependent senescence in human glioma cells. Int J Oncol 41 (5), 1669–1674. 10.3892/ijo.2012.1604 22922739

[B105] Song W. Wei L. Du Y. Wang Y. Jiang S. (2018). Protective effect of ginsenoside metabolite compound K against diabetic nephropathy by inhibiting NLRP3 inflammasome activation and NF-κB/p38 signaling pathway in high-fat diet/streptozotocin-induced diabetic mice. Int Immunopharmacol 63, 227–238. 10.1016/j.intimp.2018.07.027 30107367

[B106] Song X. Wang L. Fan D. (2022). Insights into Recent Studies on Biotransformation and Pharmacological Activities of Ginsenoside Rd. Biomolecules 12 (4), 512. 10.3390/biom12040512 35454101 PMC9031344

[B107] Stangou M. Alexopoulos E. Papagianni A. Pantzaki A. Bantis C. Dovas S. Economidou D. Leontsini M. Memmos D. (2009). Urinary levels of epidermal growth factor, interleukin-6 and monocyte chemoattractant protein-1 may act as predictor markers of renal function outcome in immunoglobulin A nephropathy. Nephrology (Carlton) 14 (6), 613–620. 10.1111/j.1440-1797.2008.01051.x 19143943

[B108] Stehouwer C. D. (2004). Endothelial dysfunction in diabetic nephropathy: state of the art and potential significance for non-diabetic renal disease. Nephrol Dial Transplant 19 (4), 778–781. 10.1093/ndt/gfh015 15031329

[B109] Su W. Y. Li Y. Chen X. Li X. Wei H. Liu Z. Shen Q. Chen C. Wang Y. P. Li W. (2021). Ginsenoside Rh1 Improves Type 2 Diabetic Nephropathy through AMPK/PI3K/Akt-Mediated Inflammation and Apoptosis Signaling Pathway. Am J Chin Med 49 (5), 1215–1233. 10.1142/S0192415X21500580 34049473

[B110] Sun Q. Meng Q. T. Jiang Y. Xia Z. Y. (2012). Ginsenoside Rb1 attenuates intestinal ischemia reperfusion induced renal injury by activating Nrf2/ARE pathway. Molecules 17 (6), 7195–7205. 10.3390/molecules17067195 22692243 PMC6268105

[B111] Sun Q. Meng Q. T. Jiang Y. Liu H. M. Lei S. Q. Su W. T. Duan W. N. Wu Y. Xia Z. Y. Xia Z. Y. (2013). Protective effect of ginsenoside Rb1 against intestinal ischemia-reperfusion induced acute renal injury in mice. PLoS One 8 (12), e80859. 10.1371/journal.pone.0080859 24324637 PMC3851764

[B112] Surh Y. J. Lee J. Y. Choi K. J. Ko S. R. (2002). Effects of selected ginsenosides on phorbol ester-induced expression of cyclooxygenase-2 and activation of NF-kappaB and ERK1/2 in mouse skin. Ann N Y Acad Sci 973, 396–401. 10.1111/j.1749-6632.2002.tb04672.x 12485900

[B113] Tam D. N. H. Truong D. H. Nguyen T. T. H. Quynh L. N. Tran L. Nguyen H. D. Shamandy B. E. Le T. M. H. Tran D. K. Sayed D. Vu V. V. Mizukami S. Hirayama K. Huy N. T. (2018). Ginsenoside Rh1: A Systematic Review of Its Pharmacological Properties. Planta Med 84 (3), 139–152. 10.1055/s-0043-124087 29329463

[B114] Tang M. Wang W. Cheng L. Jin R. Zhang L. Bian W. Zhang Y. (2018). The inhibitory effects of 20(R)-ginsenoside Rg3 on the proliferation, angiogenesis, and collagen synthesis of hypertrophic scar derived fibroblasts *in vitro* . Iran J Basic Med Sci 21 (3), 309–317. 10.22038/ijbms.2018.19451.5153 29511498 PMC5817175

[B115] Tsutsumi Y. M. Tsutsumi R. Mawatari K. Nakaya Y. Kinoshita M. Tanaka K. Oshita S. (2011). Compound K, a metabolite of ginsenosides, induces cardiac protection mediated nitric oxide via Akt/PI3K pathway. Life Sci 88 (15-16), 725–729. 10.1016/j.lfs.2011.02.011 21338613

[B116] Venkatachalam M. A. Griffin K. A. Lan R. Geng H. Saikumar P. Bidani A. K. (2010). Acute kidney injury: a springboard for progression in chronic kidney disease. Am J Physiol Renal Physiol 298 (5), F1078–1094. 10.1152/ajprenal.00017.2010 20200097 PMC2867413

[B117] Vinh L. B. Lee Y. Han Y. K. Kang J. S. Park J. U. Kim Y. R. Yang S. Y. Kim Y. H. (2017). Two new dammarane-type triterpene saponins from Korean red ginseng and their anti-inflammatory effects. Bioorg Med Chem Lett 27 (23), 5149–5153. 10.1016/j.bmcl.2017.10.058 29100799

[B118] Wada J. Makino H. (2013). Inflammation and the pathogenesis of diabetic nephropathy. Clin Sci (Lond) 124 (3), 139–152. 10.1042/CS20120198 23075333

[B119] Weiyun Z. liu F.-g. male H. t. (2019). Changes of contents of three rare ginsenosides in the process of black production %J. Shi Zhen National medicine and national medicine 30 (02), 325–327.

[B120] Wu C. Y. Hua K. F. Hsu W. H. Suzuki Y. Chu L. J. Lee Y. C. Takahata A. Lee S. L. Wu C. C. Nikolic-Paterson D. J. Ka S. M. Chen A. (2020). IgA Nephropathy Benefits from Compound K Treatment by Inhibiting NF-κB/NLRP3 Inflammasome and Enhancing Autophagy and SIRT1. J Immunol 205 (1), 202–212. 10.4049/jimmunol.1900284 32482710

[B121] Wu J. Jiang C. Hua Y. Liu X. You C. (2021a). Association between polymorphisms of cytokine genes and diabetic nephropathy: A comprehensive systematic review and meta-analysis. Int J Clin Pract 75 (11), e14634. 10.1111/ijcp.14634 34309136

[B122] Wu W. J. Tang Y. F. Dong S. Zhang J. (2021b). Ginsenoside Rb3 Alleviates the Toxic Effect of Cisplatin on the Kidney during Its Treatment to Oral Cancer via TGF-β-Mediated Mitochondrial Apoptosis. Evid Based Complement Alternat Med 2021, 6640714. 10.1155/2021/6640714 33510805 PMC7826210

[B123] Xie X. S. Liu H. C. Zuo C. Deng Y. Fan J. M. (2008a). The effect of ginsenoside Rg1 on the renal interstitial fibrosis of UUO rat. Sichuan Da Xue Xue Bao Yi Xue Ban 39 (2), 218–222. 18630687

[B124] Xie X. S. Yang M. Liu H. C. Zuo C. Li Z. Deng Y. Fan J. M. (2008b). Influence of ginsenoside Rg1, a panaxatriol saponin from Panax notoginseng, on renal fibrosis in rats with unilateral ureteral obstruction. J Zhejiang Univ Sci B 9 (11), 885–894. 10.1631/jzus.B0820024 18988308 PMC2579952

[B125] Xie X. S. Liu H. C. Li H. J. Fan J. M. (2008c). Ginsenoside R(g1) inhibit transdifferentiation in rat renal tubular epethelial cells induced by TGF-beta1. Zhongguo Zhong Yao Za Zhi 33 (17), 2136–2141. 19066060

[B126] Xie X. S. Liu H. C. Yang M. Zuo C. Deng Y. Fan J. M. (2009). Ginsenoside Rb1, a panoxadiol saponin against oxidative damage and renal interstitial fibrosis in rats with unilateral ureteral obstruction. Chin J Integr Med 15 (2), 133–140. 10.1007/s11655-009-0133-9 19407952

[B127] Xing J. J. Hou J. G. Ma Z. N. Wang Z. Ren S. Wang Y. P. Liu W. C. Chen C. Li W. (2019). Ginsenoside Rb3 provides protective effects against cisplatin-induced nephrotoxicity via regulation of AMPK-/mTOR-mediated autophagy and inhibition of apoptosis *in vitro* and *in vivo* . Cell Prolif 52 (4), e12627. 10.1111/cpr.12627 31094028 PMC6668974

[B128] Xu X. Lu Q. Wu J. Li Y. Sun J. (2017). Impact of extended ginsenoside Rb1 on early chronic kidney disease: a randomized, placebo-controlled study. Inflammopharmacology 25 (1), 33–40. 10.1007/s10787-016-0296-x 27853891

[B129] Xu J. L. Gan X. X. Ni J. Shao D. C. Shen Y. Miao N. J. Xu D. Zhou L. Zhang W. Lu L. M. (2018). SND p102 promotes extracellular matrix accumulation and cell proliferation in rat glomerular mesangial cells via the AT1R/ERK/Smad3 pathway. Acta Pharmacol Sin 39 (9), 1513–1521. 10.1038/aps.2017.184 30150789 PMC6289350

[B130] Xu L. Xiao S. Lee J. J. Li X. Zhao Y. (2022). Gender-Related Differences in Tissue Distribution, Excretion, and Metabolism Studies of Panaxadiol in Rats and Anti-inflammatory Study. J Agric Food Chem 70 (28), 8672–8679. 10.1021/acs.jafc.2c02618 35792078

[B131] Yang L. Zhang X. Y. Li K. Li A. P. Yang W. D. Yang R. Wang P. Zhao Z. H. Cui F. Qin Y. Yang J. H. Tao H. L. Sun T. Chen S. Yu P. H. Liu H. J. Yang C. (2019). Protopanaxadiol inhibits epithelial-mesenchymal transition of hepatocellular carcinoma by targeting STAT3 pathway. Cell Death Dis 10 (9), 630. 10.1038/s41419-019-1733-8 31431619 PMC6702205

[B132] Yao H. Jin Y. Yang J. Li L. Sun T. Shi X. Li X.-w. (2014). Conversion Rule of Rare Ginsenosides Produced from Major Ginsenosides by Confined Microiwave Promoted Degradation Method. Chemical Journal of Chinese Universities-chinese 35, 2317.

[B133] Yenisetti S. C. Manjunath M. J. Muralidhara C. (2016). Neuropharmacological Properties of Withania somnifera - Indian Ginseng: An Overview on Experimental Evidence with Emphasis on Clinical Trials and Patents. Recent Pat CNS Drug Discov 10 (2), 204–215. 10.2174/1574889810666160615014106 27316579

[B134] Yi Y. S. (2019). Ameliorative effects of ginseng and ginsenosides on rheumatic diseases. J Ginseng Res 43 (3), 335–341. 10.1016/j.jgr.2018.04.004 31308803 PMC6606827

[B135] Yokozawa T. Dong E. (2001). Role of ginsenoside-Rd in cisplatin-induced renal injury: special reference to DNA fragmentation. Nephron 89 (4), 433–438. 10.1159/000046116 11721162

[B136] Yokozawa T. Liu Z. W. (2000). The role of ginsenoside-Rd in cisplatin-induced acute renal failure. Ren Fail 22 (2), 115–127. 10.1081/jdi-100100858 10803758

[B137] Yung S. Yap D. Y. Chan T. M. (2017). Recent advances in the understanding of renal inflammation and fibrosis in lupus nephritis. F1000Res. 6, 874. 10.12688/f1000research.10445.1 28663794 PMC5473406

[B138] Zarneshan S. N. Fakhri S. Khan H. (2022). Targeting Akt/CREB/BDNF signaling pathway by ginsenosides in neurodegenerative diseases: A mechanistic approach. Pharmacol Res 177, 106099. 10.1016/j.phrs.2022.106099 35092819

[B139] Zhai J. Gao H. Wang S. Zhang S. Qu X. Zhang Y. Tao L. Sun J. Song Y. Fu L. (2021). Ginsenoside Rg3 attenuates cisplatin-induced kidney injury through inhibition of apoptosis and autophagy-inhibited NLRP3. J Biochem Mol Toxicol 35 (11), e22896. 10.1002/jbt.22896 34423507

[B140] Zhan M. Brooks C. Liu F. Sun L. Dong Z. (2013). Mitochondrial dynamics: regulatory mechanisms and emerging role in renal pathophysiology. Kidney Int 83 (4), 568–581. 10.1038/ki.2012.441 23325082 PMC3612360

[B141] Zhang L. N. Xie X. S. Zuo C. Fan J. M. (2009). Effect of ginsenoside Rgl on the expression of TNF-alpha and MCP-1 in rats with diabetic nephropathy. Sichuan Da Xue Xue Bao Yi Xue Ban 40 (3), 466–471. 19627007

[B142] Zhang J. J. Zhou Y. D. Liu Y. B. Wang J. Q. Li K. K. Gong X. J. Lin X. H. Wang Y. P. Wang Z. Li W. (2021). Protective Effect of 20(R)-Ginsenoside Rg3 Against Cisplatin-Induced Renal Toxicity via PI3K/AKT and NF-[Formula: see text]B Signaling Pathways Based on the Premise of Ensuring Anticancer Effect. Am J Chin Med 49 (7), 1739–1756. 10.1142/S0192415X21500828 34461812

[B143] Zhang D. Ji P. Sun R. Zhou H. Huang L. Kong L. Li W. Li W. (2022). Ginsenoside Rg1 attenuates LPS-induced chronic renal injury by inhibiting NOX4-NLRP3 signaling in mice. Biomed Pharmacother 150, 112936. 10.1016/j.biopha.2022.112936 35421784

[B144] Zhao A. Liu N. Yao M. Zhang Y. Yao Z. Feng Y. Liu J. Zhou G. (2022a). A Review of Neuroprotective Effects and Mechanisms of Ginsenosides From Panax Ginseng in Treating Ischemic Stroke. Front Pharmacol 13, 946752. 10.3389/fphar.2022.946752 35873557 PMC9302711

[B145] Zhao L. Zhang Y. Li Y. Li C. Shi K. Zhang K. Liu N. (2022b). Therapeutic effects of ginseng and ginsenosides on colorectal cancer. Food Funct 13 (12), 6450–6466. 10.1039/d2fo00899h 35661189

[B146] Zhou P. Zhan X. g Guo M. Guo R. Wang L. Zhang Z. Lin Z. Dong M. Dai H. Ji X. Lu H. (2019). Ginsenoside Rb1 ameliorates CKD-associated vascular calcification by inhibiting the Wnt/β-catenin pathway. J Cell Mol Med 23 (10), 8724747088–7098. 10.1111/jcmm.14611 PMC6787443 31423730

[B147] Zhou T. Sun L. Yang S. Lv Y. Cao Y. Gang X. Wang G. (2020). 20(S)-Ginsenoside Rg3 Protects Kidney from Diabetic Kidney Disease via Renal Inflammation Depression in Diabetic Rats. J Diabetes Res 2020, 7152176. 10.1155/2020/7152176 32258169 PMC7106937

[B148] Zhou S. He Y. Zhang W. Xiong Y. Jiang L. Wang J. Cui X. Qu Y. Ge F. (2021a). Ophiocordyceps lanpingensis polysaccharides alleviate chronic kidney disease through MAPK/NF-κB pathway. J Ethnopharmacol 276, 114189. 10.1016/j.jep.2021.114189 33964361

[B149] Zhou S. Zhou Y. Yu J. Jiang L. Xiang Y. Wang J. Du Y. Cui X. Ge F. (2021b). A neutral polysaccharide from Ophiocordyceps lanpingensis restrains cisplatin-induced nephrotoxicity. Food Sci Nutr 9 (7), 3602–3616. 10.1002/fsn3.2317 34262721 PMC8269674

[B150] Zhu Y. Zhu C. Yang H. Deng J. Fan D. (2020). Protective effect of ginsenoside Rg5 against kidney injury via inhibition of NLRP3 inflammasome activation and the MAPK signaling pathway in high-fat diet/streptozotocin-induced diabetic mice. Pharmacol Res 155, 104746. 10.1016/j.phrs.2020.104746 32156650

